# Staff Perceptions of Family-Centered Care in Italian Neonatal Intensive Care Units: A Multicenter Cross-Sectional Study

**DOI:** 10.3390/children9091401

**Published:** 2022-09-15

**Authors:** Immacolata Dall’Oglio, Rachele Mascolo, Anna Portanova, Angela Ragni, Patrizia Amadio, Martina Fiori, Marco Tofani, Orsola Gawronski, Simone Piga, Gennaro Rocco, Emanuela Tiozzo, Jos M. Latour, on behalf of the FCC Italian NICUs Study Group

**Affiliations:** 1 Professional Development, Continuing Education and Research Service, Bambino Gesù Children’s Hospital, IRCCS, 00165 Rome, Italy; 2 Semi-Intensive Care Area/Unit, Bambino Gesù Children’s Hospital, IRCCS, 00165 Rome, Italy; 3 Neonatal Surgery Unit, Medical and Surgical Department of the Fetus-Newborn-Infant, Bambino Gesù Children’s Hospital, IRCCS, 00165 Rome, Italy; 4 School of Nursing and Midwifery, Faculty of Health, University of Plymouth, Plymouth PL4 8AA, UK; 5 Unit of Epidemiology, Bambino Gesù Children’s Hospital, IRCCS, 00165 Rome, Italy; 6 Centre of Excellence for Nursing Scholarship of Nursing Professional Order, Rome Nursing College, 00146 Rome, Italy; 7 School of Nursing, Midwifery and Paramedicine, Faculty of Health Sciences, Curtin University, WA 6102 Perth, Australia

**Keywords:** family centered care, neonatal, intensive care unit, nursing, healthcare professionals, quality of care

## Abstract

Family Centered Care (FCC) in Neonatal Intensive Care Units (NICUs) included family involvement in the care process of newborns and infants. Staff perceptions of FCC may influence clinical practice and management strategies in NICUs, with an impact on quality and humanization of the care. The Family-Centred Care Questionnaire-Revised (FCCQ-R) was adapted for the NICU setting, therefore the FCCQ-R@it-NICU was developed and used for the present study in 32 Italian NICUs. We calculated internal consistency using Cronbach’s alpha correlation between Current and Necessary dimensions of the scale using the Pearson correlation coefficient. Furthermore, we investigated which characteristics could influence staff perceptions of FCC in NICUs. 921 NICU professionals participated in the study. The FCCQ-R@it-NICU revealed good internal consistency (0.96) and good correlation between dimensions (*p* < 0.05). Statistical and significant differences in Current and Necessary dimensions were found and some demographic characteristics were found predictable on FCC practice. The FCCQ-R@it-NICU is a valid tool to investigate staff perceptions about FCC in NICU settings. Profession, education level and work experience seem to positively influence the perception of what is required for FCC practice within NICUs.

## 1. Introduction

Family-centred care (FCC) in Neonatal Intensive Care Units (NICUs) offers various benefits both for infants and parents in terms of improving the physical health and the emotional development of infants and their families [1,2,3]. NICU staff play an important role in promoting, improving and putting in practice the theoretical FCC principles in NICUs, like facilitating parent participation in their child’s care, providing information to families, and educating and guiding parents, so that they may actively deal with their child’s care needs [4]. Providing FCC means that professionals must recognize that the family is the constant in a child’s life, and they must exhibit an attitude and practice made of respect, collaboration, and support [5]. Therefore, FCC requires commitment and understanding by all stakeholders involved in caring for the child and the child’s family [6,7]. Furthermore, hospital policies should encourage FCC in NICUs by including its principles in the institution’s practice standards and guidelines, and by removing barriers against the presence of families and promoting education for health professionals in this field [8].

Family-centred care in the NICU integrates parent participation in care and decision-making of their infant’s treatment and well-being. It is based on several principles, namely sharing concrete and honest information, respecting peculiarities of family-infant dyad and promoting collaboration among family and healthcare professionals. Another fundamental approach in NICU settings is the so-called Family-Centred Developmental Care that has been defined as a set of several interventions aimed to decrease the stress of preterm infants in NICUs [9]. These interventions are designed to allow optimal neurobehavioral development of the infant. Environmental, behavioural and relationship-based intervention are integrated into an individualized approach known as the Newborn Individualized Developmental Care and Assessment Program (NIDCAP) that demonstrated efficacy and good perception by healthcare professionals within NICUs [10,11]. Over the last decades, FCC has gradually been introduced into clinical practice [5,7,12,13] and has been a roadmap for NICUs [14] to facilitate the healthcare team’s ability to work with families, and meet the needs of infants and their families in this complex setting.

Staff attitudes towards FCC was explored by several authors describing the practices and perceptions of FCC by professionals in both neonatal and paediatric settings [13,15,16,17,18]. There appears to be a gap between knowledge and practice [1,16,19,20]. Therefore, staff should be encouraged to implement FCC with the awareness that this improves the health status of infants and families and parent satisfaction with the care provided. Several studies have found that staff and patient evaluations concur with regards to quality of care [8,16,19,21,22].

In Italy, NICU staff work in settings that differ across hospitals and geographical areas. The evaluation of staff perceptions about the extent FCC concepts are provided in everyday care and their necessity is important to improve quality of care, parent satisfaction and standardize FCC at a national level [16,19]. Dissonance between what is perceived as important and what is actually implemented on a daily basis in terms of FCC marks an area of frustration and dissatisfaction for both families and health professionals [5,17,18].

Differences in the way the importance of FCC is perceived and implemented by health professionals may also vary significantly across different countries and cultures [23]. In Italy, for instance, discussions about the implementation of FCC in daily practice started only in the 1980s, when children’s hospitals allowed parents to visit their children on a 24/7 basis, whereas in the UK, FCC began to be implemented 30 years earlier, in the 1950s after the Platt Report [24]. This prompted us to investigate which FCC activities health professionals consider necessary for their everyday practice. Therefore, it is important to investigate the gap between the FCC activities that are currently implemented in clinical practice and those perceived to be truly necessary [25,26]. Thus, the aims of this study are: (1) to explore and describe Italian NICU health professionals’ perceptions of the FCC concepts they consider necessary to implement and what is perceived to be actually practiced in the NICU; (2) to analyse the associations between FCC principles, the socio-demographic and job characteristics of health professionals, and the NICU organizational characteristics.

## 2. Materials and Methods

### 2.1. Study Design and Ethics

Following the STrengthening the Reporting of OBservational studies in Epidemiology (STROBE) checklists, a cross-sectional multicenter observational study was conducted in 32 Italian NICUs from January to December 2015. The NICUs in Italy are organized in both NICU and Intermediate Care Neonatal Intensive Unit and these two settings were involved in the present study. The study protocol was approved by the Institutional Review Board of the coordinating centre (prot. 828_OPBG_2014). Furthermore, every participating unit received a similar approval by the local IRB. Participation was voluntary and anonymity was ensured, and participants were assured that only aggregated data would be published. The study participants completed an informed written consent form.

### 2.2. Participants

Healthcare professionals (registered nurses, physicians, therapists, and psychologists) from 32 Italian NICUs who had previously participated in a multicentre survey were enrolled [27].

Since this was a convenience sample, we invited all the healthcare professionals that were present at work throughout the data collection period and expected an acceptance rate of at least 50%. All participants were informed that participation was voluntary and that they could withdraw from the study at any time.

### 2.3. The Family-Centred Care Questionnaire-Revised (FCCQ-R)

The Italian version of the FCCQ-R questionnaire consisting of 45 items using a Likert scale ranging from 1 “strongly disagree” to 5 “strongly agree”. It included the following domains: (1) the family as the constant of the child’s life; (2) collaboration between parents and health professionals; (3) recognize family individuality; (4) share complete information; (5) understand the developmental needs of the child; (6) encourage parent-to-parent support; (7) provide emotional and financial support; (8) assuring that the healthcare delivery system responds to family needs; and (9) provide emotional support to staff [27], as originally developed by Bruce & Ritchie [28].

This was further adapted to the NICU setting after obtaining permission from the authors of the original questionnaire. The researchers modified some items or terms that were not consistent with the NICU setting. The modified draft of the Italian version of the FCCQ-R was piloted with a sample of 21 of healthcare professionals, to check its fitness with the NICU setting and its face validity.

All the observations were collected, anonymized, and entered on an Excel spreadsheet. All the necessary amendments were made in keeping with the original questionnaire, leading to the final draft of the NICU adapted Italian version of the FCCQ-R questionnaire (FCCQ-R@it-NICU). The final open-ended questions were introduced to collect any additional suggestions about interventions that could improve family-centred care.

### 2.4. Data Collection

The head nurses of the participating NICUs or their delegates informed the participants about the aim, the methodology of the study, the purpose of the questionnaire, and that data would be kept confidential. They also provided a letter of presentation and an information sheet.

We used paper-based questionnaires for collecting data; this because it guarantees a higher response rate than web-based questionnaires [29]. Participants were asked to return the compiled questionnaires in a sealed envelope and posted it in a dedicated box. The average time required to complete the questionnaire was 15 min. The questionnaires were administered to participants for 30 days. All the data were collected by the local researchers and entered in a dedicated database developed by the research coordinating center. After checking the quality of the data collected locally, they were merged in a central database. Any incomplete questionnaire with more than one missing item response were removed before conducting the analyses.

### 2.5. Data Analysis

Data were summarized using frequencies and percentages for categorical variables, while mean and standard deviation (SD) was used for continuous variables. To determine statistical differences between groups for both categorical and continuous variables, we used the Chi-square test or Fischer’s exact test and the *t*-test or ANOVA test respectively. In keeping with the original study [28], questionnaires that had at least one missing data item were excluded from statistical analysis.

Internal consistency reliability for each FCCQ-R@it-NICU subscale was calculated using Cronbach’s alpha. According to the COnsensus-based Standards for the selection of health status Measurement Instruments (COSMIN) methodology [30,31,32], a minimum score of 0.70 for Cronbach’s alpha was considered as good. To analyze correlation between each subscale of the FCCQ-R@it-NICU, the Pearson correlation coefficient was used.

The FCCQ-R@it-NICU mean and SD scores were calculated for both current and necessary scales. In line with Bruce et al. [33] all items were averaged together to provide a total score. To examine the statistical significance of the gap between current and necessary activities of the FCCQ-R@it-NICU, paired sample *t*-tests were performed for each item, subscale, and total score.

Once determining their normality distribution, associations between the FCCQ-R@it-NICU activities, the characteristics of both healthcare professionals and clinical settings were examined through *t*-test or univariate analysis of variance (ANOVA), which was conducted using Tukey’s post-hoc test. We analysed the same variables of previous study that showed association with perception of FCC [27], namely sex, age, years of experience within the hospital, profession, level of education, and work setting. In addition, we employed multiple linear regression analysis to predict characteristics of the healthcare professionals that could impact the FCC perception assessed by FCCQ-R@it-NICU. Total score for current and necessary scales of FCC using as independent variables those with univariate *p* values < 0.20. Data were analysed using SPSS Version 22 (Armonk, NY, USA: IBM Corp).

## 3. Results

### 3.1. Participants

**Figure 1:**
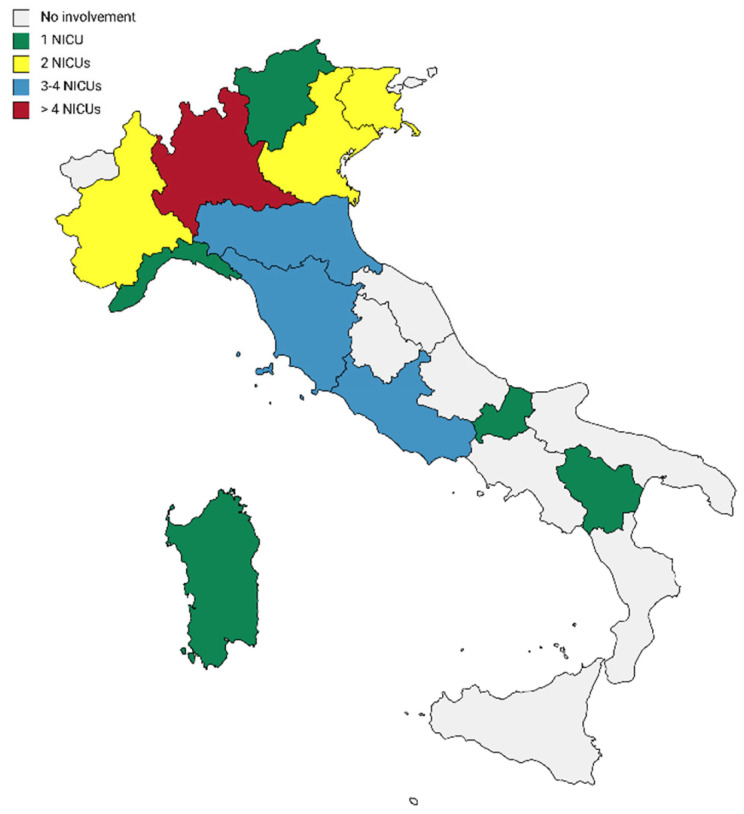
Distribution of the NICUs participating in the study.

33 NICUs in the whole Italian territory participated in the study, and most of the NICUs were from northern Italy. A graphical representation of NICUs distribution is reported in Figure 1.

A total of 960 health care professionals completed the questionnaire. The average number of professionals who completed the questionnaire for each centre was approximately 33 (SD = 17.5). Most of the professionals were females (88.3%). The sample was equally distributed in terms of age: approximately 54% of the participants were 41 years old or older; and 40% had NICU or Neonatal Intermediate Care Unit work experience between 0–5 years. Most of those participating in the research (over 91%) were full-time staff, and 5.5% had managerial and/or coordinator roles. Approximately 75% of the sample consisted of nurses, both paediatric (16.9%) and non-paediatric (57.7%), while nearly 20% were physicians. The vast majority (over 91%) worked in both NICU or Neonatal Intermediate Care Unit settings. The sample characteristics are summarized in Table 1.

### 3.2. Reliability of the FCCQ-R@it-NICU

Regarding the reliability study, the internal consistency measured with Cronbach’s alpha coefficient for Current and Necessary dimensions was 0.91 and 0.92, respectively. Values for each subscale are presented in Table 2. Furthermore, correlation between activity sub-scales of the FCCQ-R@it-NICU showed statistically significant values for both Current dimension (range 0.384–0.739) and Needed dimension (range 0.422–0.791), with a *p* < 0.05. Correlations between each activity for both FCC domains are summarized in Table 3.

### 3.3. Current and Necessary Activities of the FCCQ-R@it-NICU

Mean scores of Necessary activities showed higher values than those of Current activities of FCCQ-R@it-NICU (Table A1), highlighting that staff considered FCC activities more necessary than implemented. Regarding the differences between the current and necessary values of the FCCQ-R@it-NICU dimensions, the paired sample *t*-test also revealed significant differences for the total index (mean difference 1.05, SD 0.02) with a *p* < 0.01, as well as for each activity sub-scale. Results are summarized in Table 4.

### 3.4. Associations between FCCQ-R@it-NICU Scores and Participants’ Characteristics

Score of current, necessary and gap of the FCCQ-R@it-NICU according to participants’ characteristics revealed differences in sex, age, parental status, professions, job position and work experience. Education level revealed the main differences in each domain, namely current and necessary dimension, and in the gap. The main results are presented in Table 5.

### 3.5. Regression Model

The multivariate analysis confirmed that job profession and job position, as well as education level are independently associated with the perception of Current sub-scales of the FCCQ-R@it-NICU. Furthermore, job profession showed significant association with the perceived gap between Necessary and Current. Table 6 summarized results of statistical linear regression for Current and Necessary activities of FCC.

## 4. Discussion

The present study aimed to explore and describe Italian NICU health professionals’ perceptions of the FCC concepts they actually practiced and what they perceived as necessary to implement in NICUs. Besides, through the FCCQ-R@it-NICU we attempted to analyse the associations between FCC principles, the socio-demographic and job characteristics of health professionals.

Despite in Italy standardized tools for measuring FCC approach in Italian NICU setting are available [27,34], there is need for measuring staff perception in NICUs proved difficulties to understand how improve quality of FCC. Therefore, as outset of the present study, the research group decided to use a modified version of the FCCQ-R. The development of the Italian version of the FCCQ-R questionnaire adapted to the NICU (FCCQ-R@it-NICU) was considered mandatory because it was used in a different clinical setting, namely the NICU. The involvement of different healthcare professionals with great experience in NICUs was fundamental to adapt some items and obtain semantical equivalence with the original version. The adaptation of this questionnaire from the setting of the paediatric hospital in general to the specific setting of the NICU revealed three main categories of modification: (1) semantical: “child” was modified into “infant” because more technically appropriate for a child between birth to 23 months of age; (2) conceptual: considering the specific setting of the intervention it was not possible to directly involve infants in care process; and (3) operational: for specific operational care and procedures, items 15 and 40 were modified (see Appendix A).

With regard to reliability, Cronbach’s coefficient alpha revealed excellent internal consistency for both the current dimension (α = 0.91) and the necessary dimension (α = 0.92). This finding is in line with the original study on the FCCQ-R (0.90) [33], the Italian validation of the FCCQ-R (0.94 for both Current and Necessary dimension) [27] and the Irish version (0.93 for current and 0.94 for necessary) [35]. In particular, internal consistency was generally satisfactory, but nevertheless below the recommended standard value for some dimensions such as “Provide Emotional and Financial Support” and “the Family as the constant of the child’s life” for both Current and Necessary dimensions. These results are similar to the Italian version of the FCCQ-R [27]. The correlation between FCCQ-R@it-NICU sub-scales examined with the Pearson correlation coefficient revealed a good-to-strong relatedness for the Current dimension (range 0.558–0739) and for the Necessary dimension (range 0.605–0.791) with a strong statistical significancy (*p* < 0.01). Therefore, the present study also confirms the FCCQ-R@it-NICU as a reliable tool for measuring the approach to FCC in the target population.

Moreover, compared to the Current dimension, strong correlations were found for the Necessary dimension for each subscale. It is worth noting that for the Current we investigate the dimension of the actual status of FCC practice, while the Necessary dimension refers to what professionals considered a priority to properly implement FCC practice in NICUs. Healthcare professionals’ perspectives on current and necessary FCC approach revealed a great need to improve FCC within the NICU. In fact, the scores obtained for the Current and Necessary dimensions highlighted a statistically significant gap with a *p* < 0.001. This difference was found in each FCCQ-R@it-NICU subscale with a statistically significant difference. This finding is in line with similar studies conducted in similar contexts for both child and adult ICU [35], as well as in a non-specialised paediatric hospital in Malawi [36].

The item ‘Recognize family individuality and provide emotional support to staff’ obtained the highest scores in the Necessary dimension. This finding is in line with previous studies [28,35,36]. However, a high score for ‘Providing emotional support to staff’ was predictable, because the present study captured healthcare professionals’ perceptions.

With regard to the Current dimension, the items ‘Collaboration between parents and health professionals’ and ‘Assuring that the healthcare delivery system responds to family needs’ obtained the lowest scores. These findings are in line with a qualitative study conducted in southern Thailand in a NICU setting where the lack of collaboration between families and staff, as well as organizational and administrative issues, were found to be the main barriers for FCC in NICUs [37]. Neonatal care and nursing support for the family to participate in newborn care were significantly associated with self-efficacy; to enhance the self-efficacy of staff nurses, both the efforts of individuals and organizational strategies to promote the practice of FCC in the NICU are necessary [38]. Supporting each family member using an FCC-nursing approach is essential, especially in NICUs where the experience and behaviours of each family member facing a health situation requires a comprehensive approach to help target their unique needs [39]. Other than parents, also support for siblings should be promoted by the NICU multidisciplinary team, including nurses, social workers, physicians, and all allied healthcare professionals, to receive psychosocial support [40]. Based on institutional commitment to FCC approach, with the fundamental support of parents and organizational leadership, adequate material supports, and buy-in from staff, interventions and quality improvement projects targeting aspects of FCC in the NICU are recommended [41].

Multiple regression analysis was conducted to examine the effects of sex, type of profession, job position, age education and work experience on Current and Necessary domains, and the gap between the two domains through the FCCQ-R@it-NICU. From this analysis, it was possible to state that physicians, paediatric nurses and allied healthcare professionals had a reduction effect on this gap. Furthermore, profession and level of education influenced the perception of FCC in both the Current and Necessary domains, as well as in the gap. In addition, work experience positively influenced the perception of what is required for FCC practice within the NICU setting. Thus, our study highlighted how healthcare professionals were fully aware of the importance of parental training and visitation in the NICU to improve infant and family wellbeing, and to promote FCC.

Despite these encouraging results, our study has some limits. First of all, we did not investigate differences in participant characteristics according to the specific regional setting. Considering that the Italian national health system is regionally based and services can vary across its regions, it would be interesting to examine the differences in Current and Necessary domains for FCC practice. Another limit was the low Cronbach Coefficient’ Alpha for internal consistency for some sub-scales. Despite these results are in line with similar studies, it is worth noting that internal consistency revealed excellent values for Current and Necessary domains. In the end, another important limitation is that the data were collected in 2014, so the status of FCC practice may have changed in the meanwhile. Indeed, almost ten years later, hospital policies, structural and personnel features of Italian NICUs might have been changed. Consequently, also healthcare professionals might attribute different values to the dimensions of the questionnaire in the present day. However, being familiar with the current Italian NICU situation, we believe that no substantial changes would have occurred.

Certainly, the recent COVID-19 pandemic could have temporarily changed the priorities of the NICU healthcare staff prioritizing actions aimed at minimising the contagion rather parent participation in their child’s care. Social distancing, parent and visitor restrictions should be taken into consideration. Also, staff’s moral distress, fear for their personal and family safety might have changed the priorities of the NICU staff [42]. However, recent studies have shown how the provision of FCC could be maintained and improved even during the COVID-19 restrictions, also using mobile technology [37,43].

## 5. Conclusions

FCCQ-R@it-NICU proved to be a valid and consistent tool for use in the NICU setting and enables to capture staff perception on current and necessary practice according to the FCC framework. Work experience, education and professions may have a greater influence on staff perception; different stakeholders can now use this tool specifically developed for NICUs to improve the quality of the services and humanization of care.

After almost a decade, further research on staff perception of FCC in NICUs should be promoted considering the new global challenges, the recent pandemic, and the availability of new technical devices to help families engage in communication (i.e., smart phones for video calls, etc.).

## Figures and Tables

**Table 1:** Sociodemographic Characteristics of the Sample (n = 921).

Variables	Sample	Number	Percentage (%)
**Sex (n = 921)**			
	Female	821	(88.3)
	Male	100	(10.9)
**Staff (n = 890)**			
	Fixed-term	810	(91.01)
	Temporary	80	(8.99)
**Health Professionals (n = 916)**			
	Nurse	546	(59.61)
	Pediatric Nurse	151	(16.48)
	Medical Doctor	176	(19.21)
	Others	26	(2.84)
	HC Professional	17	(1.86)
**Job Position (n = 828)**			
	Head/Coordinator	46	(5.56)
	Non coordinator	782	(94.44)
**Education (n = 870)**			
	Regional Diploma	212	(24.5)
	Bachelor of Science	330	(37.9)
	Master of Science	66	(7.5)
	PhD or MD	152	(17.5)
	Other	110	(12.6)
**Clinical Setting (n = 914)**			
	NICU	48	(5.25)
	Intermediate Care	22	(2.41)
	Both	844	(92.34)
**Family information (n = 906)**			
	Staff with children	565	(62.36)
	Staff without children	341	(37.64)
**Age (n = 914)**			
	>51	150	(16.41)
	41–50	333	(36.43)
	31–40	304	(33.26)
	20–30	127	(13.89)
**Years of experiences (n = 437)**			
	>10	162	(37.1)
	≤10	275	(62.9)

**Table 2:** Cronbach’s Coefficient Alpha of the FCCQ-R@it-NICU.

Subscales	Current	Necessary
The family as the constant of the child’s life	0.66	0.65
Collaboration between parents and health professionals	0.76	0.70
Recognize family individuality	0.70	0.80
Share complete information	0.72	0.76
Understand the developmental needs of the child	0.65	0.75
Encourage parent-to-parent support	0.66	0.78
Provide emotional and financial support	0.57	0.61
Assuring that the healthcare delivery system responds to family needs	0.73	0.82
Provide emotional support to staff	0.74	0.85
**Total index**	**0.91**	**0.92**

**Table 3:** Correlation between each sub-scale of the FCCQ-R@it-NICU.

**Sub-Scales for Current Domains**	**1.**	**2.**	**3.**	**4.**	**5.**	**6.**	**7.**	**8.**	**9.**
1. The family as the constant of the child’s life	1								
2. Collaboration between parents and health professionals	0.639 **	1							
3. Recognize family individuality	0.426 **	0.558 **	1						
4. Share complete information	0.434 **	0.553 **	0.739 **	1					
5. Understand the developmental needs of the child	0.431 **	0.489 **	0.508 **	0.572 **	1				
6. Encourage parent-to-parent support	0.477 **	0.575 **	0.598 **	0.622 **	0.590 **	1			
7. Provide emotional and financial support	0.394 **	0.459 **	0.559 **	0.591 **	0.545 **	0.614 **	1		
8. Assuring that the healthcare delivery system responds to family needs	0.396 **	0.514 **	0.477 **	0.518 **	0.526 **	0.599 **	0.539 **	1	
9. Provide emotional support to staff	0.384 **	0.516 **	0.551 **	0.566 **	0.470 **	0.600 **	0.548 **	0.587 **	1
**Sub-Scales for Necessary Domains**	**1.**	**2.**	**3.**	**4.**	**5.**	**6.**	**7.**	**8.**	**9.**
1. The family as the constant of the child’s life	1								
2. Collaboration between parents and health professionals	0.623 **	1							
3. Recognize family individuality	0.450 **	0.605 **	1						
4. Share complete information	0.424 **	0.585 **	0.791 **	1					
5. Understand the developmental needs of the child	0.464 **	0.565 **	0.648 **	0.658 **	1				
6. Encourage parent-to-parent support	0.516 **	0.642 **	0.678 **	0.698 **	0.717 **	1			
7. Provide emotional and financial support	0.432 **	0.544 **	0.629 **	0.635 **	0.643 **	0.728 **	1		
8. Assuring that the healthcare delivery system responds to family needs	0.448 **	0.570 **	0.653 **	0.653 **	0.647 **	0.726 **	0.729 **	1	
9. Provide emotional support to staff	0.422 **	0.542 **	0.659 **	0.683 **	0.631 **	0.701 **	0.698 **	0.778 **	1

** *p* < 0.01.

**Table 4:** Gap between Current and Necessary FCCQ-R@it-NICU sub-scales.

FCCQ-R@it-NICU	Current Mean (SD)	Necessary Mean (SD)	Gap Mean (SD)	*p*-Value
The family as the constant of the child’s life	30.05 (0.84)	30.90 (0.76)	0.86 (0.87)	<0.01
Collaboration between parents and health professionals	2.79 (0.72)	3.75 (0.67)	0.97 (0.83)	<0.01
Recognize family individuality	3.39 (0.66)	4.37 (0.51)	0.98 (0.72)	<0.01
Share complete information	3.03 (0.72)	4.10 (0.59)	1.08 (0.83)	<0.01
Understand the developmental needs of the child	2.86 (0.82)	4.09 (0.67)	1.23 (0.93)	<0.01
Encourage parent-to-parent support	3.06 (0.70)	4.03 (0.61)	0.98 (0.79)	<0.01
Provide emotional and financial support	3.16 (0.70)	4.08 (0.62)	0.92 (0.76)	<0.01
Assuring that the healthcare delivery system responds to family needs	2.75 (0.67)	4.05 (0.59)	1.31 (0.85)	<0.01
Provide emotional support to staff	3.13 (0.70)	4.26 (0.56)	1.15 (0.83)	<0.01
**Total index**	**3.02 (0.20)**	**4.07 (0.18)**	**1.05 (0.02)**	**<0.01**

**Table 5:** Comparisons of FCCQ-R@it-NICU total mean score by participant characteristics.

	Current Mean (SD)	*p*-Value	Necessary Mean (SD)	*p*-Value	Gap Mean (SD)	*p*-Value
**Sex**						
Female	2.99 (0.01)	0.34	4.08 (0.48)	0.42	1.12 (0.84)	0.07
Male	3.05 (0.45)		4.04 (0.39)		0.97 (0.64)	
**Age**						
20–30	3.01 (0.52)	0.30	4.13 (0.41)	0.06	1.12 (0.83)	0.07
31–40	2.99 (0.5)		4.13 (0.51)		1.19 (0.89)	
41–50	3.00 (0.53)		4.06 (0.48)		1.11 (0.82)	
>51	3.04 (0.51)		4.02 (0.46)		0.97 (0.70)	
**Parental Status**						
With children	2.99 (0.53)	0.48	4.05 (0.48)	0.01 *	1.09 (0.85)	0.37
Without children	3.01 (0.53)		4.13 (0.45)		1.14 (0.77)	
**Professions**						
Pediatric Nurse	2.93 (0.55)	<0.01 **	4.07 (0.49)	0.02 *	1.22 (0.89)	<0.01 **
General Nurse	3.03 (0.47)		4.01 (0.52)		0.94 (0.75)	
Physician	3.13 (0.60)		4.16 (0.41)		0.97 (0.65)	
Other Allied Healthcare Professionals	3.31 (0.60)		4.19 (0.45)		0.69 (0.63)	
Others	3.22 (0.62)		4.26 (0.39)		1.12 (0.72)	
**Job Position**						
Head/Coordinator	3.20 (0.45)	<0.01 **	4.27 (0.43)	<0.01 **	1.14 (0.64)	0.89
Non coordinator	2.99 (0.53)		4.08 (0.46)		1.12 (0.80)	
**Education**						
Regional School	2.95 (0.54)	<0.01 **	3.99 (0.52)	0.002 **	1.06 (0.88)	<0.01 **
BSc	3.02 (0.53)		4.11 (0.48)		1.15 (0.84)	
MSc	2.91 (0.56)		4.17 (0.40)		1.27 (0.84)	
PhD or MD	3.17 (0.50)		4.18 (0.44)		1.05 (0.66)	
Other	2.90 (0.52)		4.07 (0.45)		1.26 (0.87)	
**Work Experience**						
Up to 10 years	3.00 (0.56)	0.92	4.13 (0.44)	<0.01 **	1.14 (0.86)	0.39
>10 years	3.00 (0.50)		4.04 (0.49)		1.09 (0.80)	
**Hospital employed**						
Yes	3.00 (0.53)	0.99	4.08 (0.47)	0.87	1.11 (0.82)	0.62
No	3.00 (0.63)		4.09 (0.54)		1.06 (0.88)	
**Work Setting**						
NICU	2.99 (0.55)	0.39	4.05 (0.49)	0.78	1.10 (0.89)	0.62
Intermediate Care	3.16 (0.66)		4.04 (0.42)		0.95 (0.80)	
Both	3.00 (0.53)		4.09 (0.48)		1.12 (0.83)	

*p < 0.05; ** *p* < 0.01.

**Table 6:** Association between Current and Necessary FCCQ-R@it-NICU and Participant Characteristics. Results of Multivariate Linear Regression.

	Current			Necessary			Gap		
	Coeff.	CI	*p*	Coeff.	CI	*p*	Coeff.	CI	*p*
**Sex**									
Male [Ref]									
Female	-	-	-	-	-	-	0.92	−0.39–2.23	0.16
**Job Profession**									
Nurse [Ref]									
Pediatric Nurse	0.73	−0.27–0.17	0.15	−0.43	−0.13–0.05	0.48	−0.13	−0.24–−0.23	**0.02**
MD	0.15	−0.66–0.37	0.17	−0.09	−0.30–0.11	0.11	−0.28	−0.53–−0.37	**0.03**
HC Professionals	0.37	0.10–0.64	**0.01**	0-13	−0.11–0.37	0.38	−0.34	−0.59–−0.11	**0.04**
Others	0.21	−0.22–0.44	0.07	0.16	−0.05–0.37	0.37	−0.69	−0.32–0.19	0.59
**Job Position**									
Staff [Ref]									
Management	0.20	0.38–0.36	**0.015**	−0.01	−0.89–0.08	0.90	-	-	-
**Education**									
Other [Ref]									
PhD/MD	0.15	−0.88–0.40	0.21	0.19	−0.03–0.42	0.08	0.10	−0.17–0.37	0.46
MSc	−0.01	−0.20–0.18	0.92	0.09	−0.07–0.27	0.26	0.16	−0.40–0.36	0.12
BSc	0.18	0.59–0.30	**0.01**	−0.01	−0.13–0.09	0.79	−0.13	−0.27–0.01	0.05
Regional Diploma	0.98	−0.03–0.22	0.12	−0.84	−0.20–0.03	0.14	−0.12	−0.26–0.19	0.09
**Age**									
>51 [Ref]									
20–30	-	-	-	0.11	−0.51–0.27	0.18	-	-	-
31–40	-	-	-	0.12	−0.01–0.23	0.05	-	-	-
41–50	-	-	-	0.05	−0.46–0.15	0.29	-	-	-
**Work Experience**									
Rif ≤ 10									
>10 years	-	-	-	−0.01	−0.90–0.79	0.79	-	-	-

## Data Availability

The datasets generated and analyzed during the present study are available from the corresponding author.

## Appendix A

**Table A1:** FCCQ-R@it-NICU Comparison between Current and Necessary Activities.

	Current	Necessary
FCCQ-R@it-NICU	Mean (SD)	Mean (SD)
**The family as the constant of the child’s life**		
1. Staff encourage parents and just in case also siblings to come and go any time that meets the family’s needs	3.30 (1.16)	4.15 (0.98)
2. Staff work with families to determine the level of participation in direct care and decision-making that suits the family’s needs best	3.50 (1.03)	4.33 (0.77)
3. The family is the key decision maker in the care of their infant	2.37 (1.00)	3.20 (1.16)
***Parent and professional collaboration***		
4. Staff determine the infant’s needs in consultation with the family and other health professionals	3.35 (1.06)	4.18 (0.85)
5. Parents contribute to the development and review of hospital policies and practices	2.24 (0.99)	2.23 (0.98)
6. Parents and siblings are involved in staff continuing education Programs in various ways.	1.98 (0.98)	1.96 (0.96)
7. Educational programs (for family members) and written material convey the sense that families are the key actors in care.	2.82 (1.05)	2.80 (1.05)
8. The admission process is used, when possible, as an opportunity to begin involving the family as members in the health care team. [if possible]	3.50 (1.05)	3.48 (1.05)
9. Hospital facilities, policies, and procedures foster family choices for participation.	2.92 (1.10)	2.90 (1.09)
10. Interviews with families are conducted in a private location.	3.12 (1.31)	4.57 (0.70)
11. Explanations are presented to the family, using a variety of techniques depending on the individual needs and learning styles of the family	3.58 (1.08)	4.52 (0.67)
12. Staff are aware of and take approaches that address the fact that families take time to develop trust.	3.77 (0.82)	4.45 (0.60)
13. Staff discuss with the family what helps them deal with events during hospitalization	3.68 (0.85)	4.40 (0.66)
14. Staff assess the level of understanding and skills of the family before and after teaching.	3.78 (0.87)	4.44 (0.60)
***Sharing information with parents***		
15. Staff promote, when possible, the infant’s preadmission information program, through which families familiarize with hospital ward staff, routines, and equipment prior to a scheduled admission to the PICU.	2.40 (1.08)	3.82 (0.97)
16. After an emergency admission or unscheduled admission, there is an organized system for helping families understand and adjust to the health care experience.	2.94 (1.10)	4.22 (0.70)
17. Information is routinely communicated to help families understand each aspect of care that their infant will experience such as anticipated sequence of events, reasons for change/treatment/procedure, ways to cope.	3.50 (0.95)	4.38 (0.68)
18. Any family member significantly involved in the infant care is encouraged to discuss or chart information about the care of their infant.	2.80 (1.04)	3.79 (0.98)
19. Staff coordinate the flow and sequence of information given to families, ensuring that the infant’s needs are apriority.	3.57 (0.94)	4.29 (0.69)
***Parent-to-parent support***		
20. Staff encourage parents to discuss concerns with other parents with similar experiences informally or in formal parent groups.	3.16 (1.09)	4.11 (0.88)
21. Staff provide programs and support for parents, siblings and members of the extended family to assist families in managing needs	2.94 (1.09)	4.19 (0.78)
22. Staff assess the needs and concerns of siblings.	2.44 (1.09)	3.78 (0.98)
23. There is a designated comfortable area for parents to gather.	2.97 (1.35)	4.29 (0.78)
***Developmental needs***		
24. Staff help family to establish/stay in touch with their family and significant others	2.68 (1.04)	3.71 (0.92)
25. Direct care managers have an adequate knowledge in infant development to support hospital staff in the practice of family-centred care.	3.44 (1.12)	4.29 (0.72)
26. Hospital brochures accurately describe the health care experience for infant and their families.	3.08 (1.12)	4.27 (0.75)
27. Staff maintain familiar routines for each infant and family.	2.56 (1.01)	3.71 (0.94)
28. Staff assess the infant interaction with staff and family.	3.56 (0.91)	4.15 (0.75)
***Emotional and financial support for families***		
29. When possible, the same staff are assigned to care for the infant and family.	3.05 (1.19)	3.93 (1.00)
30. Information and support are provided to help families understand the illness process and the impact on the infant and family, choices and risks, services, and roles of various health professionals	3.61 (0.91)	4.36 (0.69)
31. Staff recognize the financial strain on families and assist them in obtaining help	3.40 (1.00)	4.06 (0.80)
32. During procedures, a staff member is designated to explain to the family exactly what is happening.	2.64 (1.06)	3.98 (0.84)
***Design of health care system***		
33. Outpatient services are available daily and in the evening hours.	2.10 (1.04)	3.62 (1.03)
34. All written material for families is available in Italian or in the mostly commonly spoken languages	3.33 (1.11)	4.19 (0.76)
35. A written summary of relevant information about the infant is available in the primary official language of the family.	2.40 (1.12)	4.08 (0.82)
36. The physical layout of the unit is designed to meet the developmental and psychosocial needs of infant and family	2.32 (1.12)	4.24 (0.86)
37. Resources exist to help provide families with the appropriate support that they need at varying times	2.86 (1.04)	4.13 (0.78)
38. Parent evaluations are considered reliable sources of information about how well the hospital meets their needs.	3.29 (0.93)	3.99 (0.80)
39. Staffing patterns are planned according to the developmental and psychosocial needs of infant.	2.98 (1.10)	4.08 (0.84)
***Emotional support for staff***		
40. There are guidelines to assist staff in providing care during painful procedures such as: implementing pain-relieving techniques and ways to keep the child calm during and after the procedure	4.11 (0.92)	4.56 (0.65)
41. Job descriptions and performance appraisal systems incorporate expectations of family-centred care such as: knowledge of family dynamics and skills in collaboration, problem solving and interventions that address family’s needs	2.90 (0.97)	3.96 (0.76)
42. Continuing education programs provide opportunities for staff to learn to approach families effectively	3.06 (1.0)	4.23 (0.75)
43. The hospital recognizes and rewards staff’s specific knowledge and skills that are needed to care for infants and families	2.17 (1.04)	4.21 (0.87)
44. Staff are encouraged to identify, plan and evaluate new programs, policies and procedures to improve the quality of infant and family care.	3.11 (1.11)	4.32 (0.70)
45. Staff are able to express confidentially to those responsible for assuring quality care, their concerns related to those demands of providing care for their infants and families	3.40 (1.06)	4.26 (0.67)
